# Alterations of Functional Connectivity During the Resting State and Their Associations With Visual Memory in College Students Who Binge Drink

**DOI:** 10.3389/fnhum.2020.600437

**Published:** 2020-12-01

**Authors:** Bo-Mi Kim, Myung-Sun Kim, June Sic Kim

**Affiliations:** ^1^Department of Psychology, Sungshin Women’s University, Seoul, South Korea; ^2^Research Institute of Basic Sciences, Seoul National University, Seoul, South Korea

**Keywords:** binge drinking, neural oscillation, functional connectivity, theta, lower alpha, upper alpha, visual memory, Rey-Osterrieth complex figure test

## Abstract

This study investigated the characteristics of neural oscillation and functional connectivity (FC) in college students engaging in binge drinking (BD) using resting-state electroencephalography (EEG). Also, the associations of visual memory, evaluated by the Rey-Osterrieth Complex Figure Test (RCFT), and neural oscillation with FC during the resting state were investigated. The BD (*n* = 35) and non-BD (*n* = 35) groups were selected based on scores of the Korean version of the Alcohol use disorders (AUDs) Identification Test and the Alcohol Use Questionnaire. EEG was performed for 6 min while the participants rested with eyes closed. The theta, lower-alpha, and upper alpha powers did not differ between the BD and non-BD groups. Concerning FC, the BD group exhibited stronger theta coherence than that of the non-BD group, and in the lower and upper alpha bands, the BD group showed stronger coherence in some areas but weaker coherence in others compared with the non-BD group. However, these significant results were not observed after Bonferroni correction. The BD group showed significantly lower delayed recall scores on the RCFT than did the non-BD group. A positive correlation between the left prefrontal-parietal-occipital midline connection and performance on the delayed recall of the RCFT was observed in the BD group. The present results could suggest that binge drinkers have alterations in brain FC, which may be related to their visual memory deficits.

## Introduction

Binge drinking (BD), defined as drinking excessive amounts of alcohol over a short time followed by a period of abstinence, is the most common pattern of drinking among college students (Courtney and Polich, 2009). College students who engage in BD reportedly experience a variety of difficulties, including academic difficulties, drunk driving, and unsafe sexual behavior (Haller et al., 2010; Hingson and White, 2014). Additionally, binge drinkers and patients with alcohol use disorder (AUD) show similar neurological changes and cognitive impairment (Scaife and Duka, 2009; Petit et al., 2013; Winward et al., 2014), and BD during the college years may predict the development of AUDs in future (O’Neill et al., 2001).

Chronic alcohol intake (Moselhy et al., 2001) and BD (Hartley et al., 2004) cause impairments in brain structure and function. Understanding the effects of BD on the brain among college students is important to prevent future development of AUDs (Rangaswamy and Porjesz, 2014), as alcohol consumption during early adulthood, when the frontal lobe is still developing, is more detrimental to the brain than alcohol consumption at later times (Arnett, 2005; Spear, 2013).

Electroencephalography (EEG) studies during the resting state have recently been conducted to investigate the effects of alcohol on the brain (Coutin-Churchman et al., 2006; Herrera-Díaz et al., 2016; López-Caneda et al., 2017). Neural oscillations are an important mechanism of neural networks (Buzsáki and Draguhn, 2004), and changes in neural oscillations during the resting state are reportedly associated with consciousness, cognition, behavior, personality, psychopathologies, and neurological diseases (Davidson, 2003; Kumari et al., 2004; Thatcher et al., 2005). In particular, Kounios et al. (2008) found that resting-state brain activity was related to brain activity during cognitive tasks. They suggested that neural oscillations during the resting state are associated with cognitive functions such as attention, memory, and problem-solving.

Several studies have observed alterations in theta and alpha activities during the resting state in patients with AUDs (Kaplan et al., 1985; Pollock et al., 1992; Rangaswamy et al., 2003; Ehlers and Phillips, 2007). Furthermore, alpha activity is decreased in patients with AUDs compared with normal controls (Kaplan et al., 1985; Ehlers and Phillips, 2007; Mumtaz et al., 2016). However, reports of theta activity in AUD patients have been inconsistent; some studies have observed increased theta power (Pollock et al., 1992; Rangaswamy et al., 2003), whereas others found decreased theta power, in patients with AUDs (Coutin-Churchman et al., 2006) compared with normal controls.

Although studies examining the resting-state EEG activity of binge drinkers (Courtney and Polich, 2010; Correas et al., 2015; López-Caneda et al., 2017) are limited, binge drinkers showed neural oscillations similar to those observed in patients with AUDs. That is, binge drinkers had increased theta power (Correas et al., 2015; López-Caneda et al., 2017) and decreased alpha power (Correas et al., 2015) compared with the non-BD group. Also, in a study examining event-related theta power of binge drinkers and lighter drinkers, the higher the alcohol intake of binge drinkers, the lower the theta power during the visual targeting task (Correas et al., 2019). In other words, alternation of oscillation in binge drinkers was associated with worse performance on the visual targeting task.

Brain areas interact with each other to perform individual roles and continuously process information. These numerous connected brain regions form a complex and integrated network that communicates with each other linked brain areas (van den Heuvel and Hulshoff Pol, 2010). Thus, for the brain to function properly, it requires a balance between the activities of individual brain areas and the integrated activities of neural networks (Friston, 2001; Le Van Quyen, 2003). Connections of brain areas through the synchronization of neural oscillations generated in several areas are necessary to integrate this information (Buzsáki and Draguhn, 2004). In particular, it has been suggested that synchronization between more distant areas, even more than that between closer areas, is an important mechanism for communication and integrating information among brain areas (Coullaut-Valera et al., 2014). For example, a study that measured brain activity while monkeys were performing a visual memory task reported that the link between the inferior temporal cortex and the prefrontal cortex was important for recalling stored information and forming new memories (Fuster, 1997). This result indicates that activation of neural networks covering a broad range of areas is necessary to form memories *via* integration of information (Goldman-Rakic, 1995; Fuster, 1997; Magnussen, 2000). Measures of functional connectivity (FC) assess interaction among neural assemblies (Lachaux et al., 1999; Correas et al., 2015). Information spreading across various areas of the brain is constantly integrated through FC (Mheich et al., 2015); resting-state FC, in particular, may be related to the processing of complex cognitive functions (van den Heuvel and Hulshoff Pol, 2010).

FC between brain areas can be damaged by substance abuse (Sutherland et al., 2015). Especially, studies have reported that alcohol may have harmful effects on FC (Kaplan et al., 1985; Winterer et al., 2003; Correas et al., 2016; Herrera-Díaz et al., 2016) because it can impair the development of myelin, which plays an important role in the speed of information transmission (Jacobus et al., 2009; Squeglia et al., 2015). White matter damage caused by improper myelination (Hommer et al., 2001; O’Neill et al., 2001) results in impaired connections between brain areas located at a distance from each other, in turn reducing the efficiency of information transmission (de Bruin et al., 2004; Thayer et al., 2013; Correas et al., 2015). Therefore, alcohol not only damages various brain areas (Laakso et al., 2000; Males, 2009) but also impairs the connections among them.

Reduced frontal lobe (Pfefferbaum et al., 1997; Males, 2009) and white matter (Hommer et al., 2001; O’Neill et al., 2001) volumes and impaired FC (Kaplan et al., 1985; Winterer et al., 2003; Herrera-Díaz et al., 2016) have been observed in patients with AUDs. However, studies investigating FC in patients with AUDs during the resting state have obtained inconsistent findings (Kaplan et al., 1985; Michael et al., 1993; Winterer et al., 2003; Herrera-Díaz et al., 2016). For example, Herrera-Díaz et al. (2016) reported decreased alpha connectivity in the frontal-central and occipital-parietal areas in patients with AUDs relative to normal controls, whereas Winterer et al. (2003) reported increased alpha connectivity in the left temporal-parietal area in patients with AUDs compared with normal controls.

Damage to various brain areas has also been observed in binge drinkers (Courtney and Polich, 2009; Squeglia et al., 2012; Petit et al., 2013). Especially, damage to the white matter in the prefrontal and temporal lobes and the corpus callosum has been observed in binge drinkers (McQueeny et al., 2009; Smith et al., 2017), suggesting possible impairment of FC among these areas in binge drinkers. However, some studies have indicated that BD in college students could cause FC changes even before anatomical changes occur (Correas et al., 2016; Sousa et al., 2019). For example, binge drinkers showed increased brain connectivity than the control group in the default mode network, even though no significant difference in the structural connectivity was observed between groups (Correas et al., 2016).

Neural activity during the resting state is believed to be related to the performance of cognitive tasks (Kounios et al., 2008; Deco and Corbetta, 2011). In particular, theta bands during the resting state influence memory storage and recall *via* their involvement in the organization of information (Klimesch, 1999; Lisman and Jensen, 2013). Klimesch (1996, 1997, 1999) also reported that theta-band activity is involved in short-term memory and upper alpha band activity in long-term memory. Also, retrieval of stored information requires the transfer of that information among various brain areas, such as the frontal, parietal, and temporal lobes (Summerfield and Mangels, 2005). Although little is known about how various brain areas interact in this process, theta band connectivity between the hippocampus and neocortex affects the maintenance of new information (Summerfield and Mangels, 2005; Fell and Axmacher, 2011).

Several studies have reported that patients with AUDs (Rupp et al., 2006) and binge drinkers (Sneider et al., 2013; Vinader-Caerols et al., 2017) have memory deficits. In particular, studies using the Rey-Osterrieth Complex Figure Test (RCFT; Rey, 1941; Osterrieth, 1944), which evaluates visual memory, have consistently found poor performance on the immediate and delayed recall conditions of the RCFT in patients with AUDs (Dawson and Grant, 2000; Daig et al., 2010; Paikkatt et al., 2014) and binge drinkers (Hartley et al., 2004; Squeglia et al., 2009; Winward et al., 2014).

Neural oscillation during the resting state seems to be related to visual memory performance. For example, a study investigating the relationship between neural oscillation during the resting state and visual memory performance in healthy adults found that performance on a visual memory task involving immediate recall of a city map was correlated with increased theta and upper alpha powers, and performance on the delayed recall test was correlated with increased alpha power (Reichert et al., 2016). Also, performance on the copy condition of the RCFT was positively correlated with resting-state alpha power in the right frontal lobe and negatively correlated with resting-state alpha power in the left frontal lobe in patients with obsessive-compulsive disorder (Shin et al., 2004). However, no relationship between visual memory performance and neural oscillation during the resting state in binge drinkers has been reported.

Given this background, the present study investigated the effect of BD on local brain areas and the connectivity among them using resting-state EEG, spectral analysis, and brain FC analysis. Also, we investigated the relationships between neural oscillation during the resting state and visual memory performance in college students who engaged in BD.

## Materials and Methods

### Participants

The Korean version of the Alcohol Use Disorder Identification Test (AUDIT-K; Barbor et al., 1992; Lee et al., 2000) and a questionnaire inquiring about the amount/frequency of BD in the last 2 weeks were administered to 697 students using webhard. The BD and non-BD groups were defined based on: (1) alcohol-related problems and drinking habits; (2) the number of BD episodes; and (3) drinking speed.

The World Health Organization (2000) recommends that problem drinkers be identified based on a total AUDIT score ≥8 (Barbor et al., 1992). However, others have suggested a score of 12 as the most sensitive and specific cut-off for screening BD, as a score of eight indicates problem drinking even when one has no problem with alcohol currently (Kim, 1999; Lee et al., 2000). A score of 26 or higher indicates suspected alcohol dependence (Kim et al., 1999).

Therefore, in this study, those who: (1) had an AUDIT-K score >12 and <26; (2) had drunk four (female) or five (male) glasses in one sitting during the last 2 weeks; and (3) drank more than two (female) or three (male) glasses per hour were included in the BD group. Those who: (1) scored ≤8 points on the AUDIT-K; (2) had not drunk four (female) or five (male) glasses in one sitting during the last 2 weeks; and (3) drank less than two (female) or three (male) glasses per hour were included in the non-BD group (Barbor and Grant, 1989; Kim et al., 1999).

Because alcohol use can be influenced by parental alcohol use, the Korean version of the Children of Alcoholics Screening Test (CAST-K, Jones, 1983; Kim et al., 1995) was administered to identify whether the participants’ parents had a history of an AUD; those who scored ≥6 points on the CAST-K were excluded. To control for intelligence, anxiety, and depression, the Korean Wechsler Intelligence Scale (K-WAIS; Yum et al., 1992), Spielberger’s State-Trait Anxiety Inventory (STAI; Spielberger et al., 1970), and Self-Rating Depression Scale (SDS; Zung, 1965) were administered, respectively. Also, the Structured Clinical Interview for DSM-IV-Non patient edition (SCID-NP, First et al., 1996) was administered to ensure that participants had no neurological or psychiatric disorders and no drug/alcohol abuse. Only right-handed participants were included in the study.

Following the application of the initial inclusion and exclusion criteria, 45 students were placed in the BD group and 40 students were placed in the non-BD group. Since then 15 students dropped out of our procedure, ultimately, there were 35 participants (five males and 30 females, age range: 19–26 years) in the BD group and 35 participants (six males and 29 females, age range: 19–28 years) were placed in the non-BD group. Participants were instructed to abstain from using alcohol for 48 h before the experiment. All participants provided written informed consent after receiving a complete description of the study, and they were paid for their participation. This study was approved by the Institutional Bioethics Review Board of Sungshin Women’s University (SSWUIRB 2018-001).

### Rey-Osterrieth Complex Figure Test (RCFT)

The RCFT, which was developed to assess visual memory and visuoconstructional ability (Rey, 1941), consists of three conditions; copy, immediate recall (3 min after copy), and delayed recall (30 min after copy). These conditions evaluate different neuropsychological functions. The copy condition evaluates visuospatial and organizational ability, immediate recall evaluates the amount of information input and memory encoding ability and delayed recall measures information retention and retrieval (Shorr et al., 1992; Meyers and Meyers, 1995). Performance on the RCFT was scored based on Meyers and Meyers’ scoring system (1995).

### EEG Recording and Signal Processing

EEG was performed using the 64-channel Geodesic Sensor Net connected to a 64-channel high-input impedance amplifier (Net Amp 300; Electrical Geodesics, Eugene, OR, USA) in a soundproofed and electrically shielded experimental room. Resting EEG activity was recorded for 6 min in two 3-min recordings with eyes closed. All electrodes were referenced to Cz and impedance was maintained at ≤50 kΩ (Tucker, 1993). EEG activity was recorded continuously using a 0.1–100 Hz bandpass filter and a sampling rate of 500 Hz. Collected EEG data were digitally filtered using a 0.3–30 Hz bandpass filter and re-referenced to the average reference. Artifacts such as eyeblinks were removed based on the threshold of the peak-to-peak amplitude of ±100 μV from the eye channels. To conduct spectrum and FC analyses, events were selected by dividing the continuously measured EEG into 500-ms epochs and performing window sliding in each of the epochs at an interval of 250 ms. The data is displayed in units of 2 Hz in frequency, in these 500-ms epochs. For example, theta band is calculated by averaging the values of 4 Hz and 6 Hz among the data extracted at 2 Hz intervals to obtain theta band power representing the theta band (Holz et al., 2008; Anderson et al., 2010).

### Statistical Analysis

Demographic variables were analyzed using the independent *t*-test. RCFT performance was analyzed using a one-way analysis of covariance (ANCOVA) with gender as a covariate. Hanning windowing with 1 s and fast Fourier transform was used to calculate absolute power (AP) for the theta (4–7 Hz), lower-alpha (8–9 Hz), and upper alpha (10–12 Hz) bands. Mean AP for 6 min represents the magnitude of the frequency (mV^2^/Hz). To identify areal changes in frequency activity, four regions of interest (ROIs), i.e., the frontal (average of F3, Fz, and F4), central (average of C3, Cz, and C4), parietal (average of P3, Pz, and P4), and occipital (average of O1, Oz, and O2) areas were selected (Núñez-Jaramillo et al., 2015). The values of each frequency band were compared by repeated-measures mixed-design ANCOVA, using the ROIs (frontal, central, parietal, and occipital) as a within-subject factor and group (BD and non-BD groups) as a between-subject factor. The Greenhouse-Geisser correction for sphericity was employed when appropriate, and the Bonferroni correction was applied to adjust for multiple comparisons.

Imaginary coherence (ImC) was used to analyze FC. ImC, one of the most widely used methods for measuring FC in neuroscience research, can determine the degree of phase coherence between two signals measured at different electrode sites (Nolte et al., 2004; Hassan et al., 2014). The advantage of ImC is that it reduces the volume conduction problem, in that the brain activity at one source can be measured at multiple electrodes. Thus, ImC allows for observation of the actual brain activity, except for artifacts caused by volume conduction (Nolte et al., 2004). In this study, resting EEG activity was filtered by theta, lower-alpha, and upper alpha bands, and then ImC values were calculated using the following equations. First, if the Fourier transform values of two electrodes *i* and *j* for frequency *f* are *x*_i_(*f*) and *x*_j_(*f*), respectively, the cross-spectrum is defined as follows:

(1) $$S_{ij}(f)=<x_{i}(f)x_{j}^{*}(f)>$$

where * represents a complex conjugate, and < > represents an expected value. ImC was calculated by dividing the calculated cross-spectral value by the product of the power spectral values of each electrode. *f* represents frequency, *S*_ij_ represents a cross-spectrum value of electrodes *i* and *j*, and *S*_ii_(*f*) and *S*_jj_(*f*) represent power spectrum values. ImC has a value between 0 and 1, and values closer to 1 indicate full phase coherence. Fisher’s z transformation was performed on these calculated values, and the independent *t*-test was used to compare the FCs of the theta, lower-alpha, and upper alpha bands between the BD and non-BD groups. A total of 44 electrodes were analyzed after excluding the electrodes used for removing noise caused by eye or body movement (FP2, FP1, AF7, and AF8), the electrodes attached to the skin (T9, T10, P9, P10, PO7, PO8, F9, F10, 62, and 63), and the electrodes in which artifacts were recorded (F1, C1, FT7, CP5, and P1).

(2) $$ImC(f)=\frac{imaginary(S_{ij}(f))}{\sqrt{\left|S_{ii}(f)\right|\left|S_{jj}(f)\right|}}$$

The correlations between RCFT performance and FC, where the most significant difference was observed between groups, were examined using partial correlation analysis to controlling of sex. We used the Kolmogorov–Smirnov test to evaluate the normal distribution of data. Matlab version 8.3 (Math Works, Natick, MA, USA) and SPSS version 21 (IBM, Inc., Armonk, NY, USA) were used for analysis.

The correlation between fc and RCFT performance, where the most significant difference was observed between groups, was investigated using partial correlation analysis for gender control.

## Results

### Demographic Characteristics

The BD and non-BD groups did not differ in terms of age (*t*_(68)_ = −1.17, *ns*), educational level (*t*_(68)_ = 0.10, *ns*), IQ (*t*_(68)_ = −0.73, *ns*), SDS (*t*_(68)_ = 1.71, *ns*), state anxiety (*t*_(68)_ = 1.33, *ns*) and trait anxiety of the STAI (*t*_(68)_ = 1.88, *ns*). As expected, the BD group exhibited significantly higher AUDIT-K total scores (*t*_(68)_ = 24.70, *p* < 0.001), drinking speed (*t*_(68)_ = 13.42, *p* < 0.001), number of times being drunk during the previous 6 months (*t*_(68)_ = 4.92, *p* < 0.001), and percentage of times being drunk when drinking (*t*_(68)_ = 6.26, *p* < 0.001) compared with the non-BD group. The demographic characteristics of the BD and non-BD groups are presented in Table 1.

**Table 1:** Demographic characteristics of the binge drinking and non-binge drinking groups.

	Binge drinking group (*n* = 35) Mean (SD)	Non-binge drinking group (*n* = 35) Mean (SD)	*t*
Age (years)	21.31 (1.69)	21.91 (2.52)	-1.17
Years of education	15.00 (1.00)	14.97 (1.38)	0.10
IQ	112.77 (8.60)	114.29 (8.79)	-0.73
SDS	43.91 (7.09)	41.06 (6.87)	1.71
STAI state	43.63 (10.38)	40.26 (10.88)	1.33
STAI trait	46.49 (9.00)	42.40 (9.21)	1.88
AUDIT-K	19.26 (4.02)	1.11 (1.66)	24.70***
AUQ	36.90 (17.03)	4.91 (5.92)	10.50***
Drinking speed	4.34 (1.49)	0.71 (0.57)	13.42***
Number of times being drunk in the previous 6 months	9.11 (10.94)	0.03 (0.17)	4.92***
Percentage of times being drunk when drinking	52.14 (29.92)	10.57 (25.43)	6.26***

***Indicates *P* < 0.001. SD, Standard deviation; SDS, Self-Rating Depression Scale; STAI, Spielberger’s State-Trait Anxiety Inventory; AUDIT-K, The Korean version of Alcohol Use Disorder Identification Test; AUQ, Alcohol Use Questionnaire.

### Spectrum Analysis

Analyses of the theta, lower alpha, and upper alpha powers revealed significant main effects of ROI after using gender as a covariate. The theta (*F*_(1.43,95.69)_ = 18.42, *p* < 0.001), lower alpha (*F*_(1.12,74.74)_ = 18.02, *p* < 0.001), and upper alpha (*F*_(1.12,75.11)_ = 1.99, *p* < 0.001) powers were greatest in the occipital area, and lowest in the central area. However, the two groups did not differ in terms of theta (*F*_(1.43,95.69)_ = 0.12, *ns*), lower alpha (*F*_(1.12,74.74)_ = 0.46, *ns*), or upper alpha (*F*_(1.12,75.11)_ = 0.01, *ns*) power. The averaged theta, lower alpha, and upper alpha powers of the BD and the non-BD groups are presented in Table 2.

**Table 2:** Theta, lower alpha, and upper alpha power of the binge drinking and non-binge drinking groups.

	Binge drinking group (*n* = 35)			Non-binge drinking group (*n* = 35)		
	Theta	Lower alpha	Upper alpha	Theta	Lower alpha	Upper alpha
Frontal	0.34 (0.27)	0.66 (0.70)	0.29 (0.18)	0.37 (0.27)	0.95 (1.25)	0.31 (0.24)
Central	0.23 (0.14)	0.39 (0.34)	0.19 (0.10)	0.23 (0.17)	0.49 (0.57)	0.20 (0.16)
Parietal	0.28 (0.19)	0.75 (0.79)	0.41 (0.23)	0.30 (0.26)	1.00 (1.20)	0.41 (0.33)
Occipital	0.53 (0.52)	1.77 (2.21)	0.71 (0.55)	0.45 (0.35)	2.09 (3.00)	0.71 (0.92)

*( )Standard deviation*.

### Functional Connectivity

**Figure 1:**
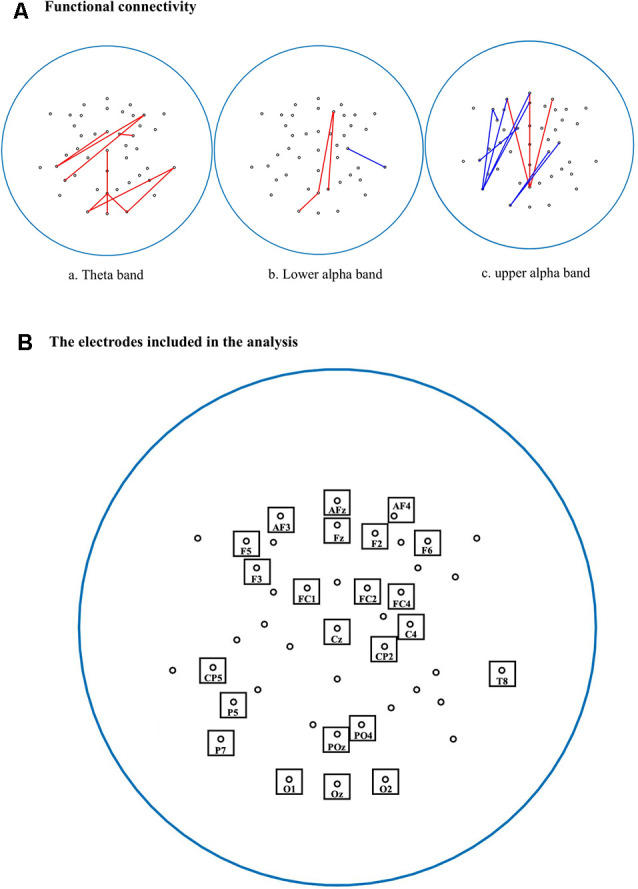
**(A)** Comparisons of functional connectivities between binge drinking (BD) and non-binge drinking (non-BD) groups. Red lines represent stronger functional connectivity (FC) in the BD group than the non-BD group, and blue lines represent weaker FC in the BD group than the non-BD group. (a) Pairs of connected electrodes in theta bands; (b) lower alpha bands; and (c) upper alpha bands (uncorrected *p* < 0.01). **(B)** The 44 electrodes were used for FC analysis. The electrodes with significant differences between groups in connectivity were marked by a box with the name of the electrode.

The estimated imaginary coherence values were normalized by Fisher’s Z-transform to compare between BD and non-BD groups. Resting-state connectivity between pairs of 44 electrodes (1,128 electrode pairs) was analyzed. Figure 1 shows the pairs of connected electrodes in which significant differences between the BD and non-BD groups were observed. Stronger and weaker connectivities in the BD group compared with the non-BD group are shown as red and blue lines, respectively (uncorrected *p* < 0.01).

In terms of the theta bands, the BD group showed stronger connectivity than the non-BD group, mainly for the right frontal-left temporal and right temporal-occipital electrode pairs. Especially, the right temporal-left occipital connectivity was strongest (*t*_(68)_ = 2.52, uncorrected *p* < 0.01).

The activity of the lower alpha band showed stronger connectivity in the right frontal-parietal-occipital midline (*t*_(68)_ = 2.40, uncorrected *p* < 0.01), parietal-occipital midline-left occipital (*t*_(68)_ = 2.53, uncorrected *p* < 0.01), and right frontal-right parietal-occipital areas (*t*_(68)_ = 2.59, uncorrected *p* < 0.01) in the BD group compared with non-BD group. On the other hand, the BD group showed weaker connectivity than that of the non-BD group in the right central-right temporal area (*t*_(68)_ = −2.54, uncorrected *p* < 0.01).

In terms of the upper alpha band, the BD group showed stronger connectivity in the right frontal-parietal-occipital midline (*t*_(68)_ = 2.51, uncorrected *p* < 0.01), prefrontal midline-parietal-occipital midline (*t*_(68)_ = 2.93, uncorrected *p* < 0.01), and left prefrontal-parietal-occipital midline (*t*_(68)_ = 2.86, uncorrected *p* < 0.01) compared with the non-BD group. However, the BD group showed weaker connectivity in the left frontal-left frontal area (*t*_(68)_ = −3.00, uncorrected *p* < 0.01) compared with the non-BD group. However, there was no significant result after applying the Bonferroni correction in the theta, lower alpha, upper alpha band.

To reduce the number of comparisons, resting-state connectivity was also analyzed using four ROIs (frontal, central, parietal, and occipital) as those used in the spectral analysis. The activity of the theta bands showed stronger connectivity in the right frontal-right parietal (*t*_(68)_ = 2.55, uncorrected *p* < 0.01), central midline-occipital midline (*t*_(68)_ = 2.49, uncorrected *p* < 0.01) and right parietal-occipital midline (*t*_(68)_ = 2.47, uncorrected *p* < 0.01) in the BD group compared with the non-BD group. In terms of the upper alpha band, the BD group showed stronger connectivity in the right frontal-left frontal (*t*_(68)_ = 1.81, uncorrected *p* < 0.05) and left frontal-left parietal areas (*t*_(68)_ = 1.78, uncorrected *p* < 0.05) compared with the non-BD group. However, there was no significant result after applying the Bonferroni correction in the theta and upper alpha band.

### Rey-Osterrieth Complex Figure Test (RCFT)

The BD group performed significantly poorer on the delayed recall (*F*_(1,68)_ = 118.49, *p* < 0.05) of the RCFT than did the non-BD group. Table 3 shows the mean scores of the copy, immediate recall, and delayed recall conditions of the RCFT for the BD and non-BD groups.

**Table 3:** (RCFT) scores of the binge drinking and non-binge drinking groups.

	Binge drinking group (*n* = 35) Mean (SD)	Non-binge drinking group (*n* = 35) Mean (SD)	*F*
Copy	32.13 (2.47)	33.11 (1.68)	17.55
Immediate recall	21.01 (5.34)	22.01 (4.86)	67.26
Delayed recall	20.30 (5.41)	22.93 (4.77)	118.49*

*Indicates *P* < 0.05. SD, Standard deviation.

### Relationship Between Functional Connectivity and Rey-Osterrieth Complex Figure Test (RCFT)

**Figure 2:**
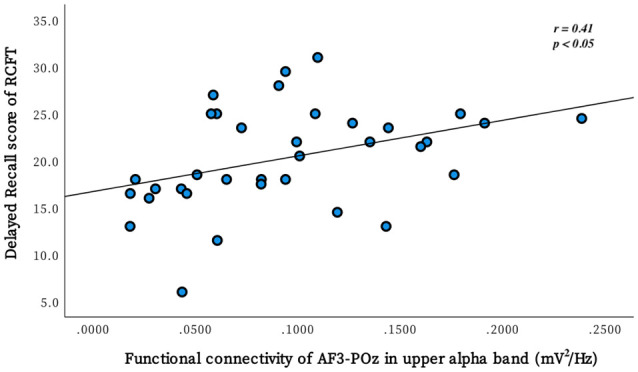
The scatter plot demonstrates the relationship between the performance of delayed recall on the Rey Complex Figure Test (RCFT) and the FC of electrodes AF3-POz in the upper alpha band. This correlation was significant in the binge drinking group but not in the non-binge drinking group.

Partial correlational analysis was conducted to investigate the associations between delayed recall performance on the RCFT and 3 connectivity (prefrontal midline-parietal-occipital midline, left prefrontal-parietal-occipital midline, and left frontal-left frontal area in the upper alpha band) where the most significant differences between the BD and non-BD groups were observed. In the BD group, a positive correlation (*r* = 0.41, *p* = 0.012) was observed between the delayed recall score on the RCFT and the connectivity of electrodes AF3-POz (left prefrontal-parietal-occipital midline; Figure 2). However, there was no significant correlation between the delayed recall score on the RCFT and connectivity in the non-BD group. There were no significant outliers, and the normal distribution of the variables was confirmed by the Kolmogorov–Smirnova test.

## Discussion

In this study, we investigated the characteristics of neural oscillation and FC in BD college students using resting-state EEG, and how these characteristics are related to visual memory. The BD and non-BD groups did not differ in terms of theta, lower-alpha, or upper alpha power.

Regarding theta power, the present results are not consistent with those of previous studies, which found increased theta power in the BD group compared with the non-BD group (Correas et al., 2015; López-Caneda et al., 2017). Theta band activity decreases as gray matter volume decreases (Squeglia et al., 2012; Howell et al., 2013; Doallo et al., 2014; Correas et al., 2015), and gray matter volume decreases gradually during normal development (Lenroot and Giedd, 2006; Fuhrmann et al., 2015). Therefore, the increased theta power in binge drinkers observed in previous studies implies delayed gray matter development in binge drinkers (Correas et al., 2015). The inconsistent findings between this and previous studies in terms of theta power may be related to participant characteristics such as age. That is, the average age and age of alcohol consumption onset of participants in previous studies were 18 and 14 years, respectively (Correas et al., 2015; López-Caneda et al., 2017), whereas those in this study were 21 and 18 years, respectively. Given that childhood drinking affects brain development, including gray matter development (Stiles and Jernigan, 2010), more negatively than does drinking at later ages, binge drinkers in this study, who started consuming alcohol later than participants in previous studies, may have been less affected by BD, which may result in maintenance of the theta power.

In the present study, the BD and non-BD groups did not differ in terms of alpha power, which is not consistent with the results of previous studies that found reduced alpha power in patients with AUDs (Krauss and Niedermeyer, 1991; Enoch et al., 1995, 1999, 2003) and in binge drinkers (Correas et al., 2015). However, reduced alpha power was observed mainly in AUD patients with anxiety symptoms (Enoch et al., 1995, 1999, 2003) or family history of AUDs (Finn and Justus, 1999), indicating that reduced alpha power may be related to anxiety and/or genetic factors. Therefore, controlling for anxiety and the genetic effects of AUDs in this study likely contributed to the nonsignificant difference in alpha power between the BD and non-BD groups.

Analysis of the FC of theta bands revealed stronger connectivity in the BD than the non-BD group. This is consistent with the results of a previous study, which reported that binge drinkers showed stronger theta connectivity than non-BD participants (Correas et al., 2015). Rapid formation and dissolution of functional connections are observed in normal people through synchronization and asynchronization between different brain areas (Herrera-Díaz et al., 2016). That is, for the brain to function properly, both synchronization and asynchronization of brain areas are required (Friston, 2000; Stam and De Bruin, 2004). The dynamics of brain FC can be impaired in two ways: by excessive connectivity or excessive disconnectivity (Stam and Van Straaten, 2012). Thus, increasing theta connectivity does not translate to better FC. For example, it has been reported that patients with epilepsy have excessive neural connections when seizures occur (Gorji and Speckmann, 2009), and patients with mild cognitive impairment show increased resting theta connectivity as a precursor to dementia (Pijnenburg et al., 2004; Buldú et al., 2011).

It has also been proposed that this abnormal hyper-connectivity is a compensatory mechanism for damaged areas or reduced or altered connectivity (Coullaut-Valera et al., 2014; Correas et al., 2015). Functional MRI studies (Squeglia et al., 2011; Xiao et al., 2013) revealed increased brain activity during cognitive tasks in BD adolescents and college students than in non-BD participants, despite similar cognitive performance levels. Thus, the increase in theta connectivity observed in the BD group may be a compensatory mechanism facilitating behavioral performance (Correas et al., 2016).

Alpha bands are involved in various cognitive processes (Klimesch, 1999; Grandy et al., 2013), and recently, the relationship between the alpha band and cognitive processes was investigated by dividing the alpha bands into lower and upper alpha bands. Specifically, lower-alpha bands play an important role in attention (Horschig et al., 2014), whereas upper alpha bands reportedly affect memory (Lebedev, 1994; Klimesch, 1997; Clark et al., 2004). The FC analysis of the lower and upper alpha bands in this study showed that the BD group exhibited stronger connections in some areas but weaker connections in other areas compared with the non-BD group.

For the lower alpha band, the BD group showed overall strong, rather than reduced, connectivity compared with the non-BD group. In terms of the upper alpha band, the BD group exhibited increased connectivity in the prefrontal-posterior parietal area. This is consistent with the results of Winterer et al. (2003), who reported increased alpha connectivity in patients with AUDs. Along with increased connectivity in the theta band, the increased connectivity in the lower and upper alpha bands may be a compensatory mechanism in response to damage in certain areas or altered connections (Coullaut-Valera et al., 2014; Correas et al., 2015).

Reduced connectivity in the upper alpha band in the BD group was observed mainly in the left frontal-left temporal and right central-left occipital areas, which is consistent with findings in binge drinkers (Correas et al., 2015), patients with AUDs (Herrera-Díaz et al., 2016), and heavy drinkers (De Bruin et al., 2006). It is suggested that reduced FC reflects weakened networks among areas (De Bruin et al., 2006; Coullaut-Valera et al., 2014); reduced FC of the alpha band may indicate damage to the structural connections among brain areas (Stam and Van Straaten, 2012). Diffusion tensor imaging (DTI) studies revealed white matter damage in binge drinkers (Jacobus et al., 2009; Smith et al., 2017). For example, McQueeny et al. (2009) reported that binge drinkers exhibit damaged white matter in the frontal, temporal, and parietal areas and the cerebellum. Thus, the decreased alpha connectivity observed in the BD group likely means that alcohol has detrimental effects on structural connections between the left frontal-left temporal and right central-left occipital areas.

The BD group performed significantly poorer on the delayed recall condition of the RCFT compared with the non-BD group. This is consistent with the results of previous studies (Scaife and Duka, 2009; Squeglia et al., 2009; Winward et al., 2014), which found visual memory deficits in binge drinkers. The delayed recall condition of the RCFT is a measure of information retention and retrieval abilities (Shorr et al., 1992; Meyers and Meyers, 1995). Hence, the present result suggests that binge drinkers may have visual memory deficits, particularly in terms of the retention and retrieval of visual information.

The correlational analysis between the delayed recall scores on the RCFT and connectivity, in which significant differences between the BD and non-BD groups were observed, was conducted to investigate the visual memory deficit observed in the BD group concerning FC. A significant positive correlation between upper alpha band connectivity in the left prefrontal-parietal-occipital midline area and delayed recall scores on the RCFT was observed in the BD group, but no significant correlation was observed in the non-BD group. The BD group showed better performance on the delayed recall of the RCFT, as the upper alpha band connectivity in the left prefrontal-parietal-occipital midline area was stronger.

Although the nervous system’s involvement in the storage and retrieval of information is not fully understood (Roudi and Latham, 2007), areas within the temporal and parietal lobes (Henson et al., 1999; Kahn et al., 2004; Wagner et al., 2005) are essential in the memory network, as activity in these areas increases during information recall (Buckner, 2004). Particularly, as the parietal lobe plays important role in visuospatial perception (Coull and Nobre, 1998; Dudkin et al., 1999), connectivity between the parietal lobes and other brain areas may be required for the storage of visuospatial information (Dudkin et al., 1999). Also, given that the geometric figures in the RCFT are complicated, successful performance on the RCFT requires organizational strategies and planning ability (Grant and Adams, 2009), which involve the prefrontal lobes (Shin et al., 2004). Thus, it could be assumed that good performance on the RCFT requires a connection between the prefrontal and parietal areas.

It has been reported that neural oscillation of the upper alpha band is associated with memory recall (Klimesch et al., 1999; Clark et al., 2004) and that better memory recall performance is associated with increased upper alpha power (Klimesch et al., 1999). However, no studies have investigated how inter-area connectivity in the upper alpha band is related to memory, despite the proposed role of the upper alpha band in memory recall.

In this study, greater upper alpha band connectivity between the prefrontal and posterior parietal areas was associated with better performance on the delayed recall condition of the RCFT in the BD group. This result could support expectations that RCFT performance requires interaction between the frontal and parietal areas and that the upper alpha connection in the frontal-posterior parietal area is involved in retrieving stored information. As increased connectivity is considered to reflect a compensatory mechanism for other damaged connections (Coullaut-Valera et al., 2014; Correas et al., 2015), the results of the present correlation analysis suggest that BD could produce detrimental effects on structural connections.

In conclusion, the BD and non-BD groups showed no significant differences in terms of theta, lower-alpha, or upper alpha activity. Concerning FC, the BD group exhibited significantly stronger theta coherence than that of the non-BD group. In terms of the lower and upper alpha bands, the BD group showed stronger connectivity in some areas but weaker coherence in others compared with the non-BD group. Also, the BD group exhibited significantly poorer performance on the delayed recall condition of the RCFT than that of the non-BD group, and a positive correlation between connectivity of the upper alpha band and performance on the delayed recall of the RCFT was observed only in the BD group. Spectrum analysis reflects the characteristics of the local brain area, whereas FC reflects the interaction among different areas. Therefore, the results of the present study indicate that BD can produce detrimental effects on the transmission of information among various areas of the brain, and this effect seems to be related to the visual memory deficits observed in binge drinkers.

This study has the following limitations. First, most of the participants were females in early adulthood, which limits the generalizability of the results to all adults. Second, as our results were not significantly derived under the multiple comparison correction, the present results should be interpreted carefully. Third, as EEG was performed only in a resting state but not during a visual memory task, direct comparisons of EEG activity in the resting state and during memory task performance were not possible. Therefore, further research in which EEG activity recorded during the memory task and resting-state is compared would allow for a clearer understanding of the default mode network and the neural mechanisms of visual memory. Fourth, this study used only spectrum and FC analyses. The application of network analysis would provide additional information about the connectivity efficiency, as well as the number of connections that show differences between groups.

## Data Availability Statement

The raw data supporting the conclusions of this article will be made available by the authors, without undue reservation.

## Ethics Statement

The studies involving human participants were reviewed and approved by Sungshin Women’s University Institutional Review Board (SSWUIRB 2018-001). The patients/participants provided their written informed consent to participate in this study.

## Author Contributions

M-SK helped in conceptualization, funding acquisition, and supervised the overall aspects of the article. B-MK contributed to formal analysis, interpretation of the results, and writing of the manuscript. JK supervised the data analysis part of the experiment. All authors reviewed the manuscript. All authors contributed to the article and approved the submitted version.

## Conflict of Interest

The authors declare that the research was conducted in the absence of any commercial or financial relationships that could be construed as a potential conflict of interest.
